# Program Cell Death Receptor-1-Mediated Invariant Natural Killer T-Cell Control of Peritoneal Macrophage Modulates Survival in Neonatal Sepsis

**DOI:** 10.3389/fimmu.2017.01469

**Published:** 2017-11-20

**Authors:** Eleanor A. Fallon, Tristen T. Chun, Whitney A. Young, Chyna Gray, Alfred Ayala, Daithi S. Heffernan

**Affiliations:** ^1^Division of Surgical Research, Department of Surgery, Brown University and Rhode Island Hospital, Providence, RI, United States

**Keywords:** invariant natural killer T cells, programmed cell death receptor-1, neonatal, sepsis, peritoneal macrophage

## Abstract

We have shown that invariant natural killer T (*i*NKT) cells mediate sepsis-induced end-organ changes and immune responses, including macrophage bacterial phagocytosis, a finding regulated by the check point protein program cell death receptor-1 (PD-1). Furthermore, PD-1 mediates mortality in both adult and neonatal murine sepsis as well as in surgical patients. Given our previous findings, we hypothesize that *i*NKT cells will also modulate neonatal sepsis survival, and that this effect is regulated in part through PD-1. We utilized a polymicrobial intra-peritoneal cecal slurry (CS) sepsis model in wild type (WT), *i*NKT^−/−^ or PD-1^−/−^ 5–7 day old neonatal pups. Typically, tissues were harvested at 24 h for various bioassays/histology and, in some cases, survival was assessed for up to 7 days. Interestingly, similar to what we recently reported for PD-1^−/−^ mice following CS, *i*NKT^−/−^-deficient animals exhibit a markedly improved survival vs. WT. Histologically, minor alterations in liver architectural, which were noted in WT pups, were attenuated in both *i*NKT^−/−^ and PD-1^−/−^ pups. Following CS, PECAM-1 expression was unchanged in the WT pups but increased in both *i*NKT^−/−^ and PD-1^−/−^ pups. In WT, following CS the emergence of a Ly6C^low^ subpopulation was noted among the influxed peritoneal macrophage population. Conversely, within *i*NKT^−/−^ pups, there were fewer peritoneal macrophages and a greater percentage of Ly6C^high^ macrophages. We show not only a key role for *i*NKT cells in affecting end-organ damage as well as alterations in phagocytes phenotypes in neonatal sepsis but that this *i*NKT cell mediated effect is driven by the central checkpoint protein PD-1.

## Introduction

Sepsis in neonates remains a devastating illness. Despite significant advances in medical and surgical ICU care, both sepsis-related mortality and long-term morbidity from residual multi-organ dysfunction remain dismally high (1, 2). Intra-abdominal sepsis, from a variety of causes, remains a leading etiology of neonatal sepsis. Several investigators aim to mimic a specific surgical disease such as necrotizing enterocolitis by including a variety of components to the models (3). However, there is a paucity of data pertaining to the isolated effect of the abdominal bacterial burden without a significant tissue damage component. Using a neonatal model, we demonstrated a role for the check point protein program cell death receptor-1 (PD-1) in modulating the mortality seen with isolated peritoneal polymicrobial sepsis (4). PD-1 is a check point protein involved in both co-stimulatory and co-inhibitory regulation of a variety of acute and chronic immune responses (4–7).

Immune dysfunction induced by sepsis is a major driver of the noted mortality and the morbidity from distant organ failure/dysfunction (8). Following sepsis patients manifest immunosuppression (9). However, the predominance of our understanding of the immune dysfunction seen in sepsis is derived from adult data, both murine models and critically ill patients. It is well recognized that considerable differences exist between the neonatal and adult immune systems. Compared to the adult immune system, it is noted that the neonatal system exhibits a predominance of the innate arm and a poorly developing cellular immunity component (10). Despite these well-described differences in naive baseline immune systems, it is critical to identify key clinical and immunological manifestations of immune suppression that are present across all ages. It will be key to understand whether pathways described in adult sepsis, for which therapeutic agents are currently available, also exist in neonatal sepsis.

We and others have previously demonstrated that several key regulators of the immune response exist. Specifically, this includes a central role for invariant natural killer T (*i*NKT) cells (11–14). *i*NKT cells are innate regulatory cells, which may modulate the immune response to polymicrobial sepsis in both adult mice and humans. This involves regulating peritoneal macrophages (11) and liver kupffer cells (15). *i*NKT cells regulate clearance of the peritoneal bacterial septic burden and affect influx of key immune cells. Specifically, within adults, we have noted that this *i*NKT cell regulation of the immune response to sepsis is controlled by the regulatory checkpoint protein PD-1 (16), a finding not previously observed in neonates.

Invariant natural killer T cells appear to be well developed early in the neonatal immune system and display a considerable non-thymic component to their development and mobilization and functioning. Furthermore, they play key roles in response to non-infectious allergic responses in the early immune system. Given this and the key role for iNKT cells in adult polymicrobial sepsis, we hypothesize that iNKT cells will play a key role in regulating neonatal response to peritoneal sepsis, a finding regulated by the checkpoint protein PD-1.

## Materials and Methods

### Mice

Wild-type (WT) mouse pups were bred from C57BL/6J parents. Mice deficient in either *i*NKT cells (to the deletion of the Vα14Jα18 T-cell receptor gene; Jα18^−/−^) or gene for PD-1 (PD-1^−/−^) breed on a C57BL/6 background were used to breed the knock-out strains. All mice were bred at Rhode Island Hospital and maintained at our institution’s rodent facility receiving standard care and standard diet. All pups used were aged 5–7 days old at the start of any experimental procedure. Research objectives and all animal protocols were approved by the Institutional Animal Care and Use Committee of Rhode Island Hospital (ACUP# 0040-16) and conducted in accordance with the Animal Welfare Act and National Institutes of Health guidelines for animal care and use.

### Cecal Slurry (CS) Model

The CS model at our institution is modification (16) of the model previously described by Wynn et al. (17). In brief, a naive WT adult donor mouse (C57BL/6J) was euthanized and cecal contents were harvested. These cecal contents were mixed with 5% dextrose solution to create a CS with a concentration of 80 mg/mL. Pups from each litter used were randomly assigned to Sham (Sh) or CS groups. This was repeated across several litters. For the CS group, pups aged 5–7 days old underwent intra-peritoneal (IP) injection of CS at an LD_70_ (1.3 mg/g BW) as a septic challenge (17). Matched pups from the same litter underwent IP injection of 0.9% saline served as the Sh control.

### Survival Study

Survival studies were undertaken comparing survival of the pups up with 7 days following either Sh or CS injection. Survival checks were undertaken four times per day for the first 3 days and then twice daily up to 7 days. Given that a striking difference in mortality (50% mortality in the WT) was evident by 24 h both in this study as well as our previous work (4), the 24 h time point was chosen for the rest of the experiments.

### Tissue Collection

Pups were euthanized at 24 h following Sh or CS *via* decapitation. They underwent peritoneal lavage for the collection of cells, which were freshly prepared for flow cytometric analysis. Liver was then harvested and stored either in formalin or at −80°C for later western blot analysis.

### Flow Cytometry

Peritoneal cells were collected by lavage, centrifuged, and analyzed fresh by flow cytometry. Cell counts were also undertaken to calculate absolute numbers of cells within each population. To identify *i*NKT cells, we used α-GalCer pre-loaded CD1d tetramers conjugated to allophycocyanin (APC) (specific for the Vα14Jα18-TCR). The control was unloaded tetramer, both of which have been obtained from the NIAID Tetramer Facility (Germantown, MD). Monoclonal antibodies were used for most analyses. These included fluorescein isothiocyanate-conjugated F4/80 (peritoneal macrophages), phycoerythrin (PE)-conjugated anti-CD69 (activation), APC-conjugated anti-Ly6C (maker of activation and transmigration ability), and APC-conjugated CD11b (macrophage activation and mobilization factor). All antibodies used were in accordance with both manufacturer’s recommendation and our previous publications and analyzed *via* FloJo software.

### Histology

Liver samples were placed in formalin and subsequently embedded in paraffin for later sectioning. Sections were stained with hematoxylin and eosin and reviewed for architecture, including ballooning and apoptosis. All H&E samples were analyzed in a fashion blinded to the strain or experiment of origin.

### Western Blot

Protein lysates of mouse hepatocytes were run on 10% Tris-glycine gels (Invitrogen, Carlsbad, CA, USA). Blotting procedures, chemiluminescent detection, and densitometric analysis were performed as previously described by our laboratory (18). Membranes were probed with PECAM-1 polyclonal antibody (cat# ab28364; Abcam, USA) and bands detected at 130 kDa. Glyceraldehyde 3-phosphate dehydrogenase was used for loading control.

### Statistical Analysis

SigmaPlot 12.5 (Systat Software, San Jose, CA, USA) was used for all analyses. Data are expressed as mean and standard error of the mean. Categorical data were assessed using chi-squared or Fisher’s exact test. Mann–Whitney *U* test was used to assess continuous data across two groups. One-way analysis of variance with Holm–Sidak *post hoc* analysis was used for continuous data across multiple groups. Alpha was set to 0.05.

## Results

**Figure 1:**
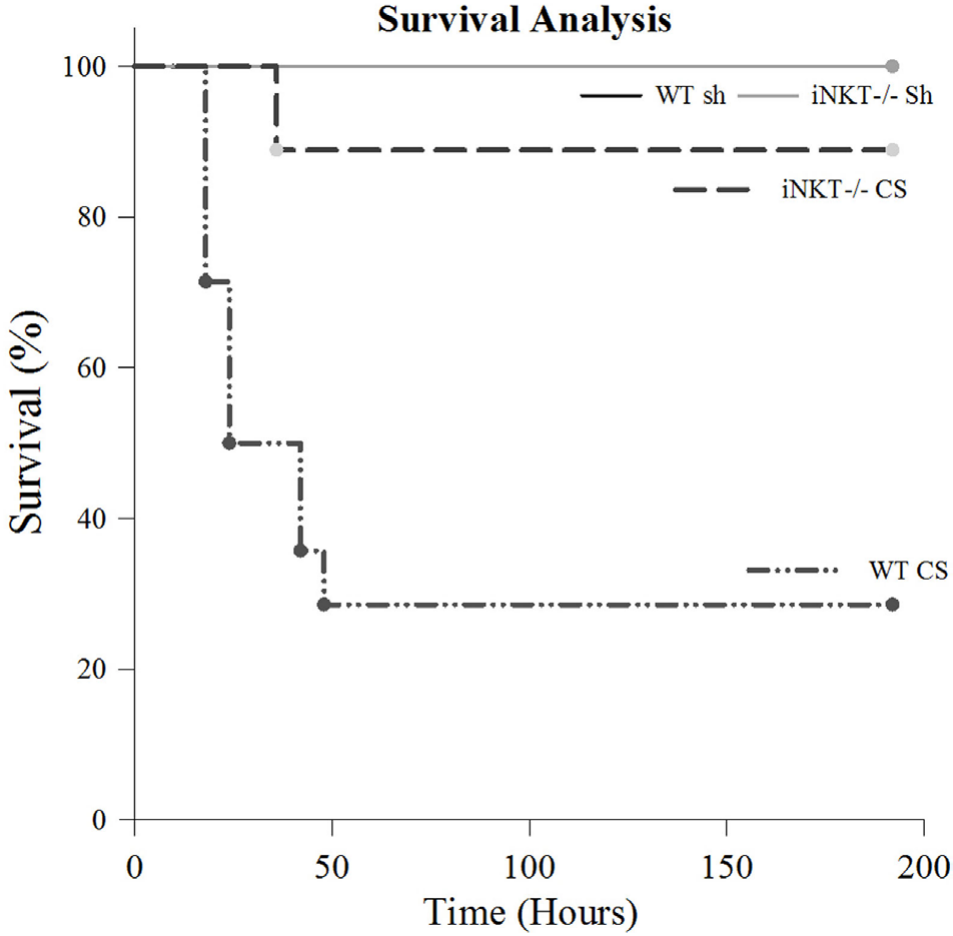
Survival study. There was no mortality among either wild type (WT) or *i*NKT^−/−^ pups subjected to sham (*N* = 7). Compared to sham, sepsis induced approximately 70% mortality among WT pups (*p* < 0.001) (*N* = 14). However, compared to WT, this mortality was considerably abrogated among *i*NKT^−/−^ pups mortality, noted to be only approximately 10% (*N* = 21) (*p* < 0.001 compared to WT).

Given our finding that *i*NKT cells play a role in modulating mortality from sepsis in adult mice (11, 13), we first undertook a survival analysis to assess for a potential role for *i*NKT cells in neonatal sepsis. Akin to previous observations in both adult as well as in neonatal sepsis (4), no mortality was noted in either WT or *i*NKT^−/−^ pups following Sh injection (*N* = 7). CS injection induced an early mortality of approximately 70% in WT pups (*N* = 14) (*p* < 0.001 compared to both Sh groups). The mortality difference was evident as early as 24 h. However, it was abrogated in *i*NKT^−/−^ pups wherein a mortality of only approximately 10% was noted (*N* = 21) (*p* < 0.05 – compared to WT CS) (Figure 1). Given the fact that mortality effects occurred as early as 24 h following CS in WT, we opted for 24 h as the time point for all further work. Specifically, we reviewed the role for *i*NKT cells in affecting both the local (peritoneal cavity) as well as remote organ (liver) responses to polymicrobial sepsis.

**Figure 2:**
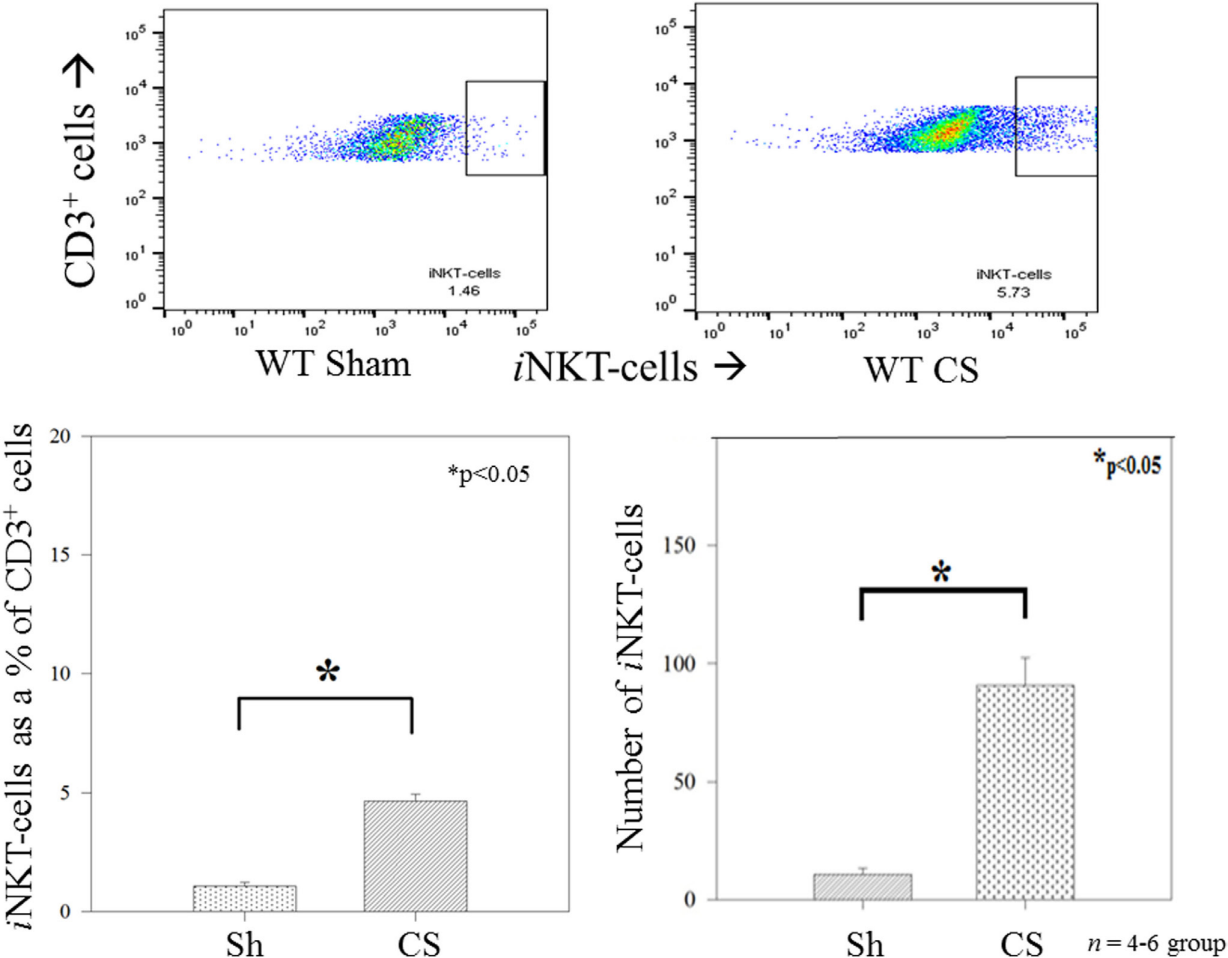
Invariant natural killer T cells were noted to appear in the peritoneal cavity following sepsis, both as a percentage of CD3^+^ cells as well as absolute numbers of cells. *N* = 4–6 per group. *p < 0.05 comparing sham to CS groups.

We have previously demonstrated a role for *i*NKT cells in regulating the peritoneal macrophage phenotype and function in response to sepsis in adults (11), we determined whether *i*NKT cells would affect changes in the neonatal peritoneal macrophage response which may begin to explain the role of *i*NKT cells in affecting mortality following neonatal sepsis. Twenty-four hours following induction of sepsis, the peritoneal cavity underwent lavage with aspiration of peritoneal cells, which were then assessed using flow cytometry. Initially, we noted that in WT pups, CS induced an influx of *i*NKT cells into the peritoneal cavity, both as a percentage of CD3^+^ cells as well as absolute numbers (Figure 2).

**Figure 3:**
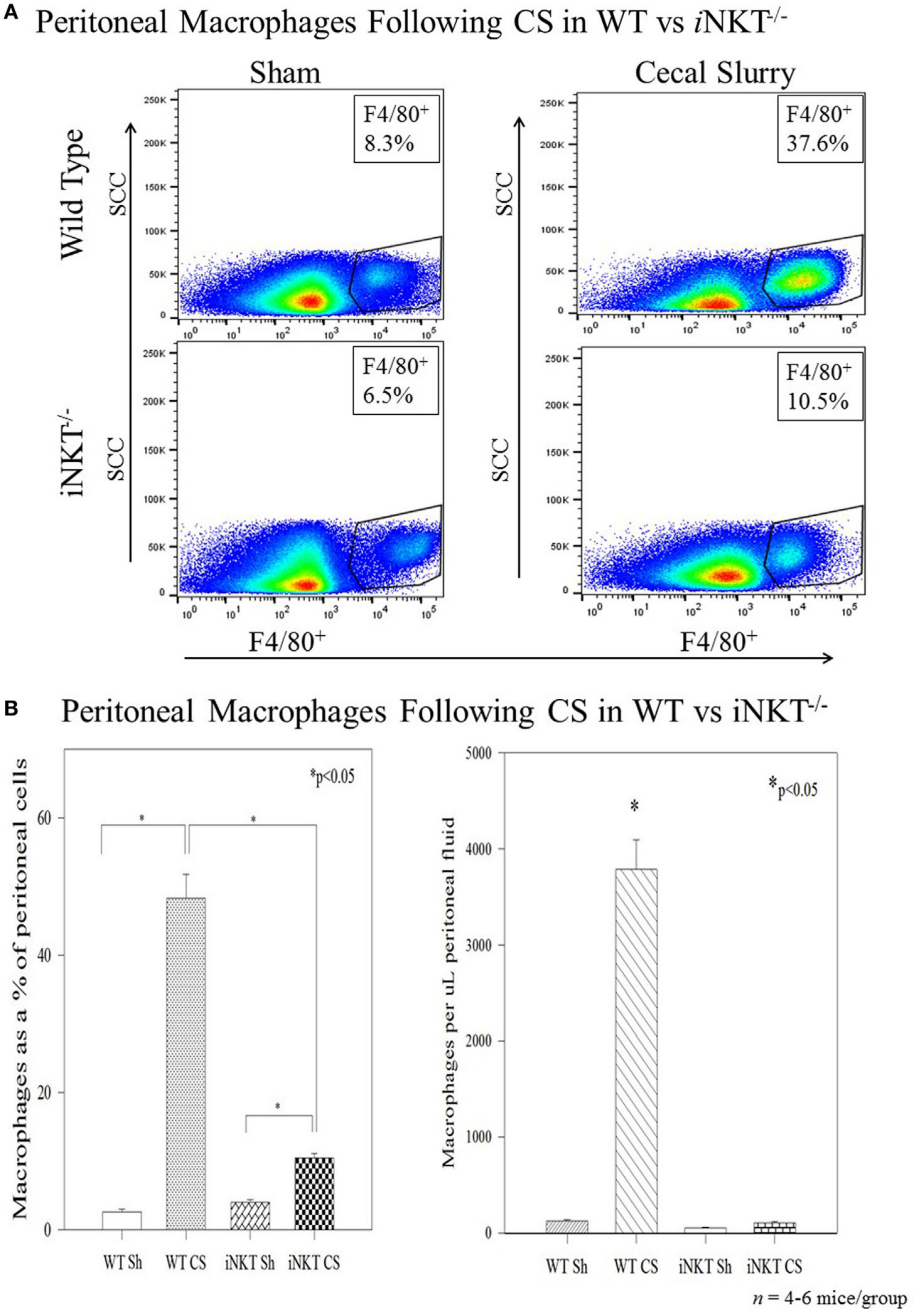
**(A,B)** Sepsis induced an influx of macrophages into the peritoneal cavity in wild-type pups but not in *i*NKT^−/−^ pups. Analysis of variance with *p < 0.05 to detect differences between each group.

Among the WT pups, compared to Sh, CS induced a marked influx of macrophages into the peritoneal cavity at 24 h both as a percentage of total peritoneal cells (3% vs. 49%; *p* < 0.01), as well as absolute numbers of cells. However, in *i*NKT^−/−^ pups, although there was a small increase in peritoneal macrophage influx, there was a marked reduction of peritoneal macrophages when compared with WT pups (Figure 3). To assess for PD-1’s role in this finding, we repeated the study among PD-1^−/−^ pups and observed a similar finding wherein CS within PD-1^−/−^ pups was not associated with a significant peritoneal macrophage influx.

**Figure 4:**
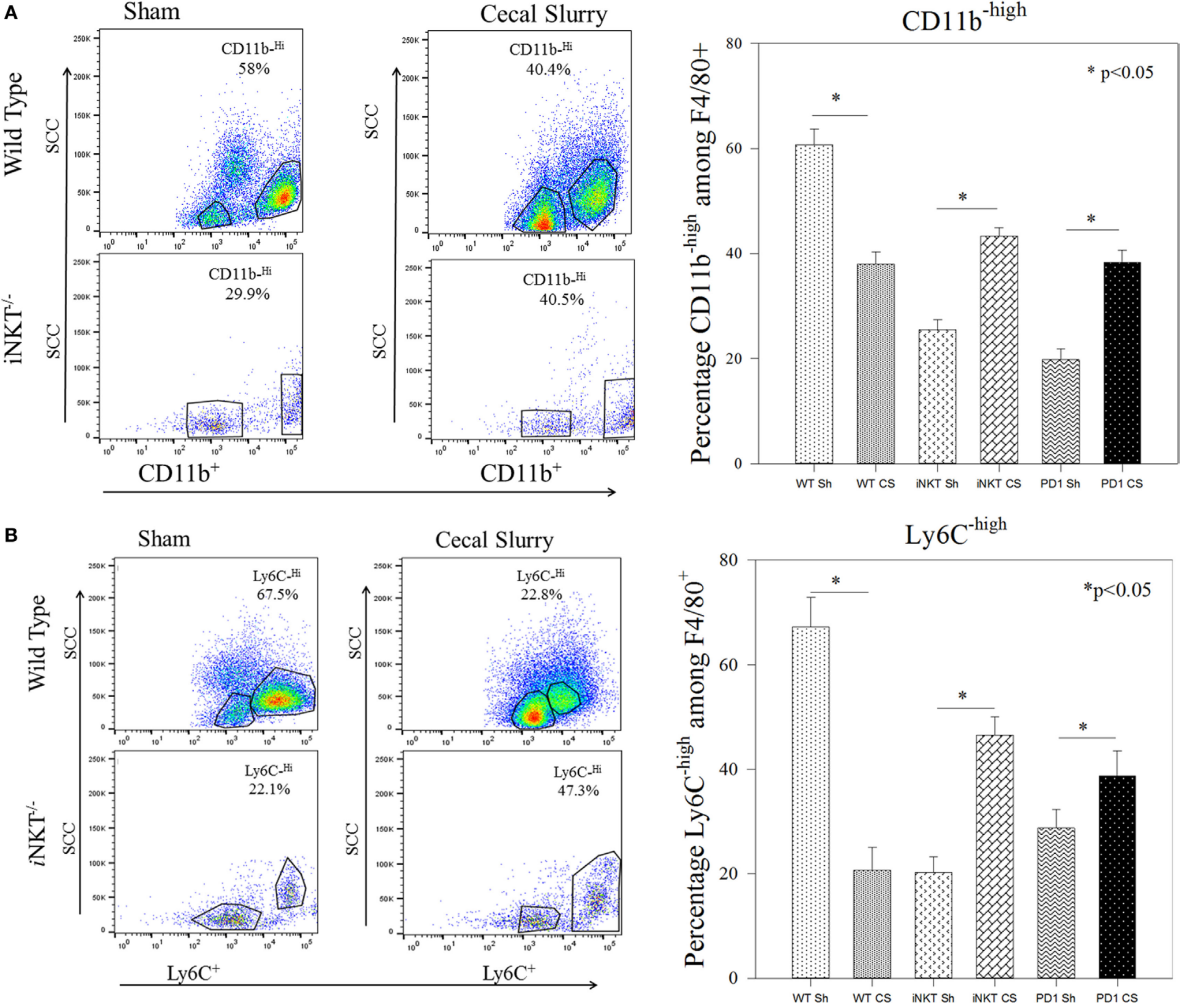
**(A,B)** Representative flow cytometry of CD11b^high^ and Ly6C^high^ phenotyping of the peritoneal F4/80^+^ macrophages. Sepsis in wild-type sepsis induced a decline of the CD11b^high^ and Ly6C^high^ sub-populations, a finding reversed in both *i*NKT^−/−^ and PD-1^−/−^ pups. *N* = 4–6 per group. Analysis of variance with *p < 0.05 to detect differences between each group.

To further determine whether *i*NKT cells played a role in altering the phenotype of these infiltrated macrophages, we undertook flow cytometric analysis for CD11b^high^ versus CD11b^low^ expression among the peritoneal macrophages. In WT, CS was noted to induce an expansion of the CD11b^low^ subpopulation (9.5% vs. 40%; *p* < 0.05) and a decline in the CD11^high^ (58% vs. 40%; *p* < 0.05) population within the peritoneal cavity. Conversely, in *i*NKT^−/−^ pups, the opposite effect was noted, wherein there was a decline in the CD11b^low^ (49% vs. 29%; *p* < 0.05) and an expansion of the CD11b^high^ subpopulation (Figure 4A). A similar effect was noted when assessing for Ly6C expression, wherein sepsis in WT induced an expansion of Ly6C^low^ peritoneal macrophages, but in *i*NKT^−/−^pups, this was reversed with an expansion of the Ly6C^high^ peritoneal macrophage subpopulation. To address a potential role of PD-1 in this observation, we also assessed a similar peritoneal macrophage phenotype in PD-1^−/−^ pups. In pups lacking PD-1, the peritoneal macrophage phenotype mimicked that found in the *i*NKT^−/−^ pups for both CD11b and Ly6C populations (Figure 4B).

**Figure 5:**
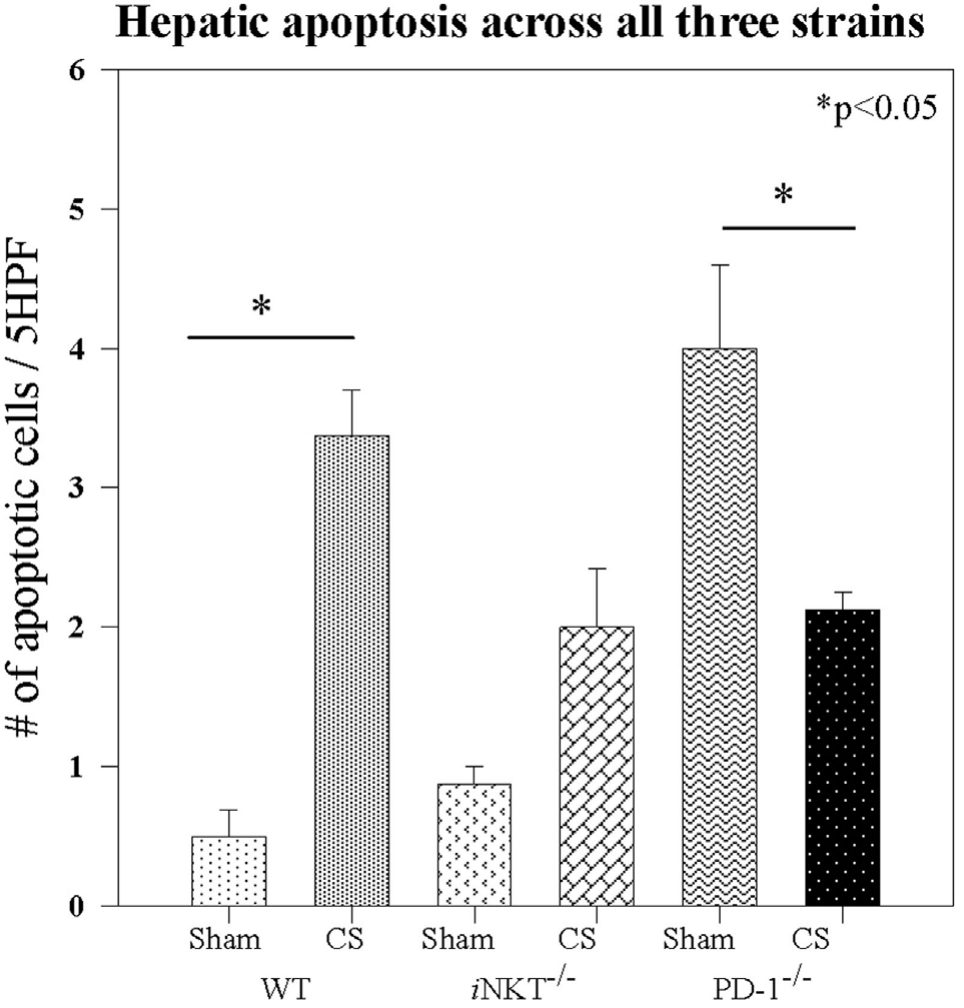
Sepsis induced apoptosis in wild-type pups, but sepsis did not increase apoptosis in either *i*NKT^−/−^ pups or PD-1^−/−^ pups. *N* = 4–6 per group. Analysis of variance with *p < 0.05 to detect differences between each group.

We next assessed whether *i*NKT cells may affect distant organ response to sepsis. Given the fact that a large contribution to the morbidity and mortality of neonatal sepsis has previously been shown to be derived from distal organ failure (19), and specifically liver dysfunction (20), coupled with the central role of hepatic *i*NKT cells in responding to both local and distal sterile and infectious insults, we next focused on whether *i*NKT cells may also contribute to potential liver damage as a contributing aspect to the noted sepsis related mortality differences. On H&E staining, it was evident that CS did not lead to any gross architectural damage or any evidence of fulminant liver architectural changes in either the WT pups or *i*NKT^−/−^ or PD-1^−/−^ pups. We next reviewed minor liver derangements that may contribute to mortality. We have previously noted within adult models that sepsis induces apoptosis, thus, we looked for apoptotic cells, per 5 high power fields, in liver specimens. Compared to Sh, CS sepsis was associated with an induction of apoptosis in the WT pups (0.5 vs. 3.8; *p* = 0.002). *i*NKT cells appeared to play a role in sepsis induced apoptosis, given that no difference in hepatic apoptosis was evident between Sh and CS in *i*NKT^−/−^ pups. To assess whether the check point protein PD-1 may play a role in this finding, we undertook a similar analysis in PD-1^−/−^ pups. There were two interesting findings within this group. First, it was noted that levels of apoptosis within Sh PD-1^−/−^ were markedly higher than had been noted within WT Sh pups. Sepsis was noted to induce comparable levels of apoptosis within the liver in both *i*NKT^−/−^ and PD-1^−/−^ pups. However, relative to Sh levels, counter to the findings in *i*NKT^−/−^ pups, we noted that when compared to Sh, rather than being increased, we note decreased apoptosis in PD-1^−/−^ pups (4.3 vs. 2; *p* = 0.01) (Figure 5).

**Figure 6:**
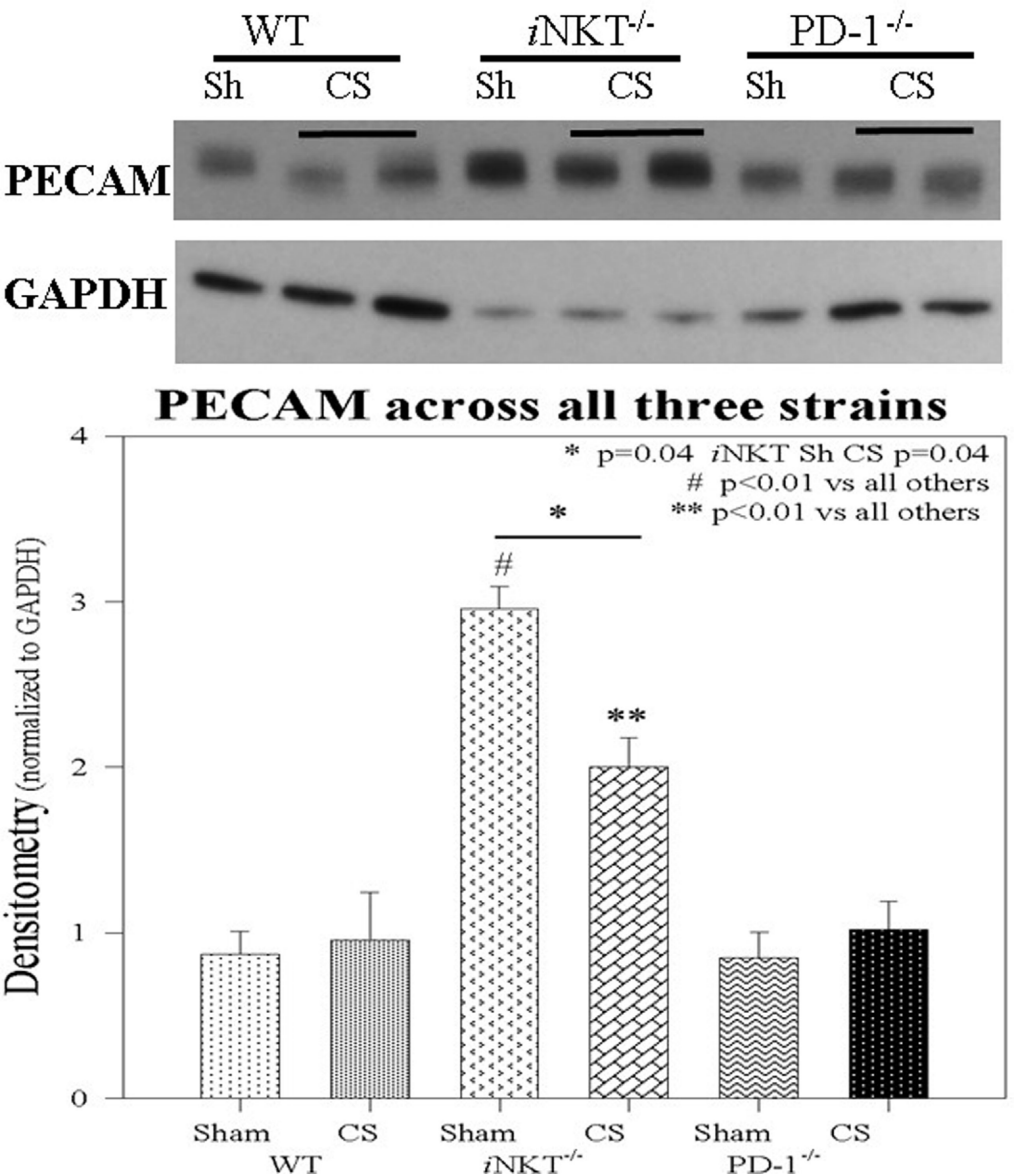
PECAM-1 expression was unchanged in either wild-type or PD-1^−/−^ pups following cecal slurry (CS). However, within *i*NKT^−/−^ pups, PECAM-1 expression was noted to be significantly elevated at baseline following sham compared to all others. Following CS sepsis, levels of PECAM were noted to significantly decrease. *N* = 4–6 per group. *p = 0.04 comparing *i*NKT^−/−^ sham versus CS; ^#^*p* < 0.01 comparing *i*NKT^−/−^ sham versus all others; **p < 0.01 comparing *i*NKT^−/−^ CS versus all others; by analysis of variance.

Given the central role for PECAM-1 in a combination of both gap junction integrity as well as a role in mediating leukocyte transendothelial migration in response to inflammation in distant organs, we assessed PECAM-1 expression in the liver. Within WT pups, CS did not induce any alteration in PECAM-1 expression. This was also noted within PD-1^−/−^ pups following sepsis. However, within *i*NKT^−/−^ pups, it was notable that the baseline level of PECAM-1 was markedly elevated compared across the other strains as well as compared with the other disease states. Furthermore, within *i*NKT^−/−^ pups, CS was noted to induce a marked decline, compared to Sh in PECAM-1 expression. However, this was still elevated when compared to the either WT or PD-1^−/−^ pups (Figure 6).

## Discussion

Sepsis from a peritoneal source in the neonate remains a devastating illness with high mortality rates and, among those who survive, high rates of long-term morbidity and residual end-organ dysfunction (2). Factors controlling the neonate’s immune response to sepsis have begun to be elucidated. Despite the marked differences that exist between the neonatal and adult immune systems, understanding which similarities will offer insights into controlling mechanism of the sepsis response across the ages. Specifically, within the neonate, it is emerging that innate regulatory cells appear to be more abundantly present. Specifically, a distinct and active population of *i*NKT cells exists in neonatal cord blood (21, 22). We have previously demonstrated a significant role for *i*NKT cells in controlling the immune response to sepsis in adults (11, 13). In this study, we echo some of those findings. We note a role for *i*NKT cells in affecting both the local peritoneal macrophage response and a distal organ (liver) response to polymicrobial abdominal sepsis. Previous authors have demonstrated some functional differences between naive neonate and adult *i*NKT cells, wherein both specific stimuli and local environment dictate type of *i*NKT cell as well as response from the *i*NKT cells from neonates compared to adults (23). However, this prior work is almost exclusively undertaken in cord blood and following stimulation of isolated naive *i*NKT cells and does not reflect a potential role for these unique regulatory cells in response to polymicrobial neonatal sepsis. Furthermore, our current work echoes a previously noted role for the check point protein PD-1 in modulating mortality from sepsis in both humans and mice as well as a role for PD-1 in modulating the adult iNKT cell response to sepsis (16). Furthermore, we recently demonstrated a role for PD-1 in survival in neonatal sepsis (16). The current work using a model of neonatal peritoneal bacterial sepsis distinct from the additive effects of tissue destruction demonstrates a role for *i*NKT cells in affecting both the local peritoneal and distant organ response to neonatal sepsis and focused on PD-1 as a possible driver of these *i*NKT-cell-mediated effects. These results offer exciting potential therapeutic targets given the clinical availability, for adults as well as pediatric patients, of both specific modulators of *i*NKT cells (24, 25) and PD-1 antagonists.

We have previously demonstrated in adult mice, following initiation of sepsis, that hepatic *i*NKT cells leave the liver and migrate to the peritoneal cavity, wherein, they have a regulatory role in controlling the peritoneal macrophage response to sepsis. As a mechanism, we demonstrated that this *i*NKT-cell migration and *i*NKT cell control of the peritoneal macrophage response was under the regulation of the checkpoint protein PD-1 (16). Within the neonate, it is known that innate regulatory immune cells, such as *i*NKT cells, play major roles in responding a variety of antigens, including the fact that *i*NKT cells are noted to accumulate in the small intestine in the second trimester (26). This supports our current observation that *i*NKT cells emigrate into the peritoneal cavity in response to sepsis and that these *i*NKT cells played a significant role in altering the peritoneal macrophage population.

Neonates are known to display an attenuated and down-regulated inflammatory response to many stimuli (8, 10, 27). Despite this dampened inflammatory response, it has been postulated that neonatal mortality may be driven from end organs being more susceptible to the immune response to an infection (8). Although we did not find any gross histologic hepatic damage following sepsis, we did detect minor alterations within the liver following sepsis. Any degree of organ dysfunction among vulnerable neonates may display marked effects; however, further work will be needed to assess whether these observed minor effects indeed play a role in altering sepsis related mortality. *i*NKT cells appear to play a role in the sepsis induced apoptosis, wherein apoptosis was diminished in Jα18^−/−^ pups following sepsis. CS induced an alteration in PECAM-1 expression in WT pups. PECAM-1 (CD31) is expressed at the lateral borders of endothelial cells. Within the fetal and neonatal liver, PECAM-1 is expressed upon endothelial cells of all blood vessels and is involved in many of the developmental and pathologic changes seen in response to disease processes (28, 29). Alterations in PECAM-1 affect vascular responses and leukocyte trafficking in response to a septic challenge (30). We herein noted that *i*NKT cells potentially maintain gap junction integrity by regulating PECAM-1, wherein *i*NKT^−/−^ pups displayed lowered hepatic PECAM-1 expression compared with the WT. This is in keeping with the observations of Clement et al who demonstrated key interactions between innate regulatory lymphocytes and cell adhesion molecules (31).

Here, we have demonstrated that several features of the peritoneal macrophage response to peritoneal polymicrobial septic challenge are controlled by *i*NKT cells in the neonate. Within WT pups, sepsis induced an influx of *i*NKT cells into the peritoneal cavity. This peritoneal influx of *i*NKT cells is in keeping with our adult murine data, in which the liver is the major source of *i*NKT cells in the mouse, and that when a septic source is detected, hepatic *i*NKT cells become activated, enter the circulation, and localize to the peritoneal cavity to affect the immune response to sepsis (11, 16). Most importantly, there was a marked decline in the influx of macrophages in the absence of *i*NKT cells; this speaks to a central role for *i*NKT cells in mobilizing macrophages to the source of the sepsis. Furthermore, we noted *i*NKT-cell-dependent alteration in the activation status of the peritoneal macrophages in response to sepsis. We chose to assess CD11b and Ly6C^high/low^ level expression among these cells. CD11b is often seen as a marker of activation of macrophages and is also involved in facilitating transendothelial migration (32, 33). The emergence of CD11b^low^ subpopulation following sepsis in the WT is in keeping with the generalized immunosuppression seen in sepsis. However, the reversal of this effect with the lack of CD11b^low^ and the expansion of CD11b^high^ among *i*NKT-cell-deficient mice is in keeping with the known role for *i*NKT cells as suppressive or anti-inflammatory immune cell. Many have described the *i*NKT-cell as the brake on the immune system to control and prevent an over-exuberant inflammatory and immune response (34, 35). Ly6C was chosen as both a marker of activation and surface receptor involved in aiding mobilization of macrophages in anticipation of possible migration (36). Again given the emergence of Ly6C^low^ in WT, but not in *i*NKT cell-deficient mice, would suggest a role for *i*NKT cells in modulating and preventing too many macrophages getting to the site of injury or sepsis and, thus, preventing an excessive response.

We believe that our data are in keeping with the known dual abilities of *i*NKT cells (14, 35, 37, 38). It has been well-described that *i*NKT cells are capable of both a Th1 response and a Th2 response to a variety of insults as well as affecting the local or distal Th1/Th2 response in adult animal models. Our data supports a model of *i*NKT cells acting as co-stimulator cells licensing macrophages to respond to a stimulus (the bacterial burden) and then mobilize and migrate to the peritoneal cavity (the source of the sepsis) as reflected by the lack of peritoneal macrophages in CS in *i*NKT cell deficient mice. However, once these macrophages are activated and arrive at the peritoneal cavity, then *i*NKT cells appear to be responsible for controlling and preventing an over-exuberant immune response and, thus, preventing tissue destruction and by-stander tissue injury.

Our findings also support the concept that the *i*NKT-cell migration and their actions directed at macrophages were under the influence of PD-1. Among immune regulators, PD-1 has arisen to play an integral role in a variety of physiologic derangements, including sepsis and malignancy (39). PD-1 is a transmembrane receptor that is recognized to serve as a checkpoint protein in both pro- and anti-inflammatory cascades. It has been demonstrated to alter cell-trafficking in response to intra-abdominal sepsis in the adult mouse model *via* release of the immunoparalysis (40, 41) induced by severe sepsis (42, 43). This includes affecting the trafficking of *i*NKT cells to the source of sepsis in adult mice (16). When PD-1 is activated *via* binding to one of its two ligands, it acts to prevent the activation of immune cells. This causes downregulation of the function of T and pro-B cells among other effects (6, 41). Although as it name suggests the molecule promotes apoptosis among antigen-specific T cells in lymph nodes, it reduces apoptosis among regulatory suppressor T cells. It also contributes to inhibition of macrophage function in the setting of sepsis (44). Conversely, when PD-1 is blocked, it cannot exert its inhibitory effects on most T cells (6). PD-1 inactivation neutralizes the pathway thought to be critical in T-cell co-stimulatory signal regulation.

This work, expanding upon an aspect of *i*NKT-cell biology, offers intriguing future potential targets for currently available therapeutic agents. *i*NKT cells are activated *via* α-galactosylceramide (αGalCer), which is a potent and very specific glycolipid. Although much of the clinical work on *i*NKT-cell and PD-1 modulation has been undertaken among cancer patients, modified αGalCer glycolipids have been used to modulate *i*NKT cells in phase I/II clinical studies involving patients with chronic hepatitis B (45) and chronic hepatitis C infections (24). Interestingly, Yang et al. demonstrated that the effectiveness of *i*NKT cells in affecting control of chronic hepatitis B was, in part, driven through PD-1 expression upon *i*NKT cells. Tefit et al. demonstrated the safety of ABX196, another αGalCer analog, as a vaccine against hepatitis B (46). Given the safety and effectiveness of these early cancer and hepatitis trials involving αGalCer glycolipid analogs, future work will be aimed at sicker and more vulnerable septic patient population, specifically as it relates to the need for higher and repeat dosing to overcome profound immunosuppression. Furthermore, an ability to direct a Th1 versus a Th2 response has been demonstrated based on specific structural analogs of αGalCer (47).

Several authors have begun to explore the ability to harness the power of donor *i*NKT cells. Specifically relating to our findings, De Lalla et al. noted that, among pediatric patients with hematological malignancies receiving hematopoietic stem cell transplantation, the successful reconstitution of *i*NKT cells was key to maintaining cancer remission (48). This work is also in keeping with our prior data among septic adult/geriatric patients wherein we noted that loss of an adequate circulating *i*NKT population following sepsis was associated with an increased risk of death (12). With the expanded abilities to grow and maintain transplantable *i*NKT cells, one can foresee septic patients receiving transplanted *i*NKT cells followed by either modified αGalCer glycolipids or CD1d blocking agents to modulate the function of these transplanted *i*NKT cells.

## Conclusion

Here, we demonstrate a key role for *i*NKT cells in affecting both peritoneal macrophages as well as end-organ damage in neonatal sepsis. This *i*NKT-cell-mediated effect is, in part, driven by the central checkpoint protein PD-1, a ligand for which therapeutic agents are currently clinically available. Given the currently available agents either in clinical use or undergoing clinical trials for the modulation of both *i*NKT cells as well as PD-1, ongoing elucidation of this mechanism will allow further refinement of potential therapeutic targets in this vulnerable population with neonatal sepsis.

## Ethics Statement

The study was carried out in accordance with the recommendations of the Rhode Island Hospital IACUC (Institutional Animal Care and Use Committee). The protocol was approved by the Rhode Island Hospital IACUC.

## Author Contributions

Design and concept: EF, TC, WY, CG, AA, and DH. Conduct of experiments: EF, TC, WY, CG, and DH. Results analysis and interpretation: EF, TC, WY, AA, and DH. Manuscript preparation, revisions, and approval: EF, TC, WY, AA, and DH.

## Conflict of Interest Statement

The authors declare that the research was conducted in the absence of any commercial or financial relationships that could be construed as a potential conflict of interest.

## Abbreviations

WT, wild type; *i*NKT, invariant natural killer T; PD-1, program cell death receptor-1; Sh, sham; CS, cecal slurry; PECAM-1, platelet endothelial cell adhesion molecule; GAPDH, glyceraldehyde 3-phosphate dehydrogenase; α-GalCer, alpha-galactosylceramide; CD, cluster of differentiation; FITC, fluorescein isothiocyanate; PE, phycoerythrin; APC, allophycocyanin; H&E, hematoxylin and eosin; kDa, KiloDalton; ANOVA, analysis of variance.
